# Are bone erosion and peripheral feeding vessels hallmarks of intracranial solitary fibrous tumor/hemangiopericytoma?^☆^

**DOI:** 10.1016/j.radcr.2022.04.050

**Published:** 2022-06-01

**Authors:** Hiroki Sugiyama, Satoshi Tsutsumi, Akane Hashizume, Toshihisa Inaba, Hisato Ishii

**Affiliations:** a Department of Neurological Surgery, Juntendo University Urayasu Hospital, Urayasu, Chiba, Japan; b Department of Pathology, Juntendo University Urayasu Hospital, Urayasu, Chiba, Japan; c Division of Radiological Technology, Juntendo University Urayasu Hospital, Urayasu, Chiba, Japan

**Keywords:** Solitary fibrous tumor/hemangiopericytoma, Cerebral convexity, Bone erosion, Mimic meningioma

## Abstract

An 86-year-old man sustained progressive motor weakness in the left lower extremity for 1 month. Cranial computed tomography (CT) revealed an isodense mass in the right parietal lobe, with a smooth-contoured focal erosion in the adjacent parietal bone. The extra-axial tumor appeared isointense on T1- and hyperintense on T2-weighted magnetic resonance imaging with intense enhancement. On three-dimensional CT angiography, the ectatic left occipital artery coursed into the right parietal foramina and connected with a dilated meningeal vessel supplying the tumor. The focal erosion formed in the inner parietal bone was located adjacent to the feeding vessel. A total tumor resection was achieved. The microscopic findings of the resected specimen were consistent with a World Health Organization grade III hemangiopericytoma (HPC). Bone erosion and peripheral feeding vessels may be characteristic findings of intracranial solitary fibrous tumor (SFT)/HPC. Careful interpretation of neuroimages could help detect clues for distinguishing an SFT/HPC masquerading as a meningioma from a true meningioma.

## Introduction

Meningiomas are a frequent brain tumor of the central nervous system. However, a variety of tumors can radiologically mimic meningiomas, which can cause confusion in presurgical diagnosis [1]. Solitary fibrous tumors (SFT) and hemangiopericytomas (HPC) are rare extra-axial tumors that have been classified as a single disease entity, that is, SFT/HPC, in the 2016 World Health Organization (WHO) guidelines. SFT/HPCs occasionally mimic meningioma but tend to lead to a worse overall prognosis and lower survival rate due to local recurrence and distant, extracranial metastasis [2,3]. The presence of bone erosion, absence of hyperostosis, sunburst flow void, and intratumoral calcification, and differing values of apparent diffusion coefficient have been proposed as radiological findings differentiating between meningiomas and SFT/HPCs [4], [5], [6], [7], [8], [9], [10]. In contrast, meningiomas accompanying bone erosion and SFT/HPCs with calcifications have also been reported [11,12].

After branching from the external carotid artery, the occipital artery initially courses deep sites from its origin to the arch formed by the trapezius and sternocleidomastoid muscles. The artery then becomes superficial while ascending to the vertex, where it occasionally penetrates the fine bony canal(s), parietal foramen, or foramina that are typically located in the parasagittal region, just anterior to the rhomboid suture, and anastomoses with the meningeal vessels [13], [14], [15], [16].

Here, we report a unique case of SFT/HPC accompanying bone erosion that was fed by the transcranial blood supply from the contralateral occipital artery.

## Case report

**Fig. 1:**
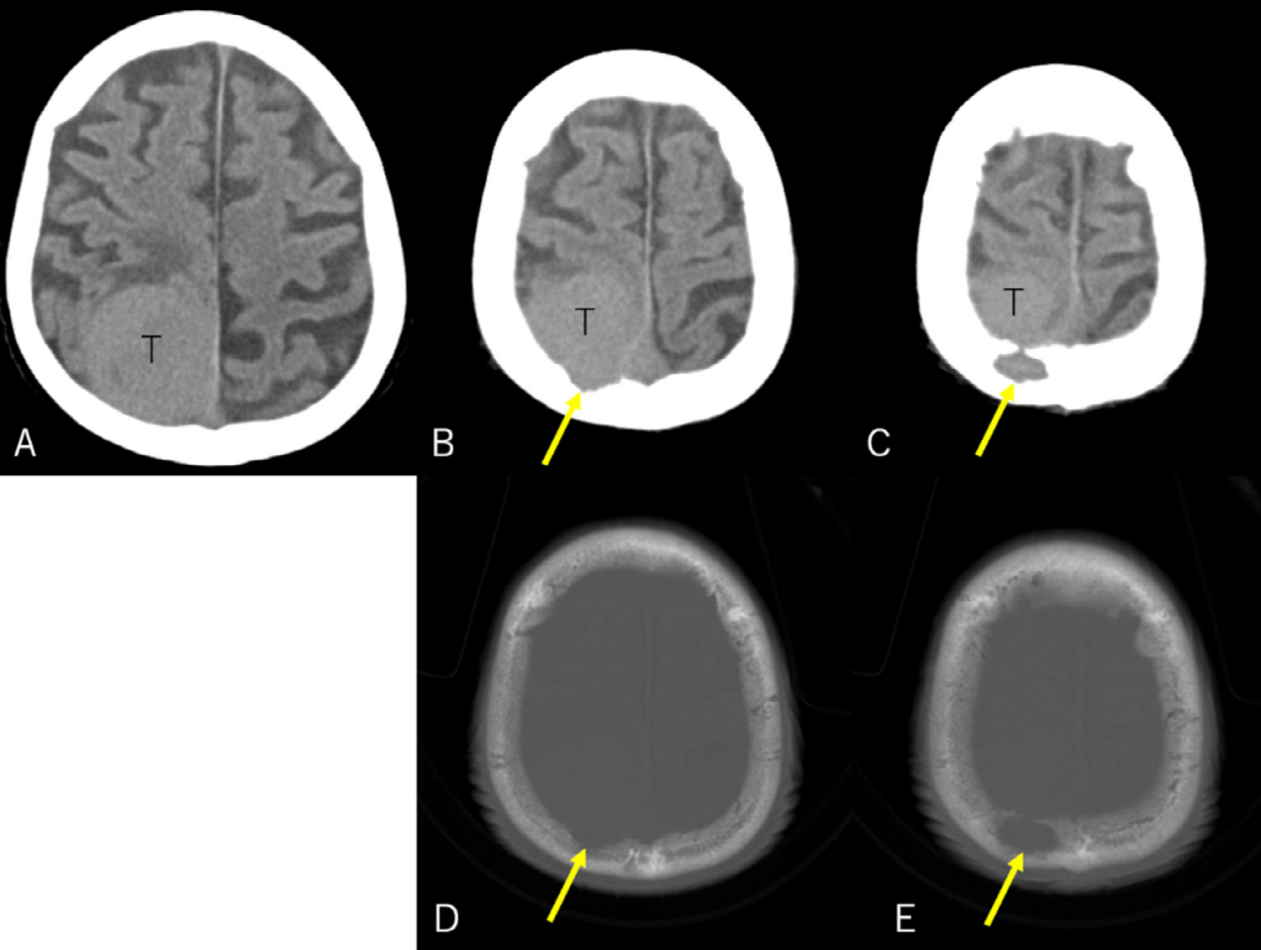
Noncontrast axial computed tomography scans (***A-C***) and relevant bone-target images (***D, E***) showing an isodense mass in the right parietal lobe with a smooth-contoured focal erosion in the adjacent parietal bone (***B-E,*** arrow). Neither intralesional calcification nor hyperostosis are observed in the adjacent skull. T: tumor. ***A*** (inferior)→***C*** (superior).

**Fig. 2:**
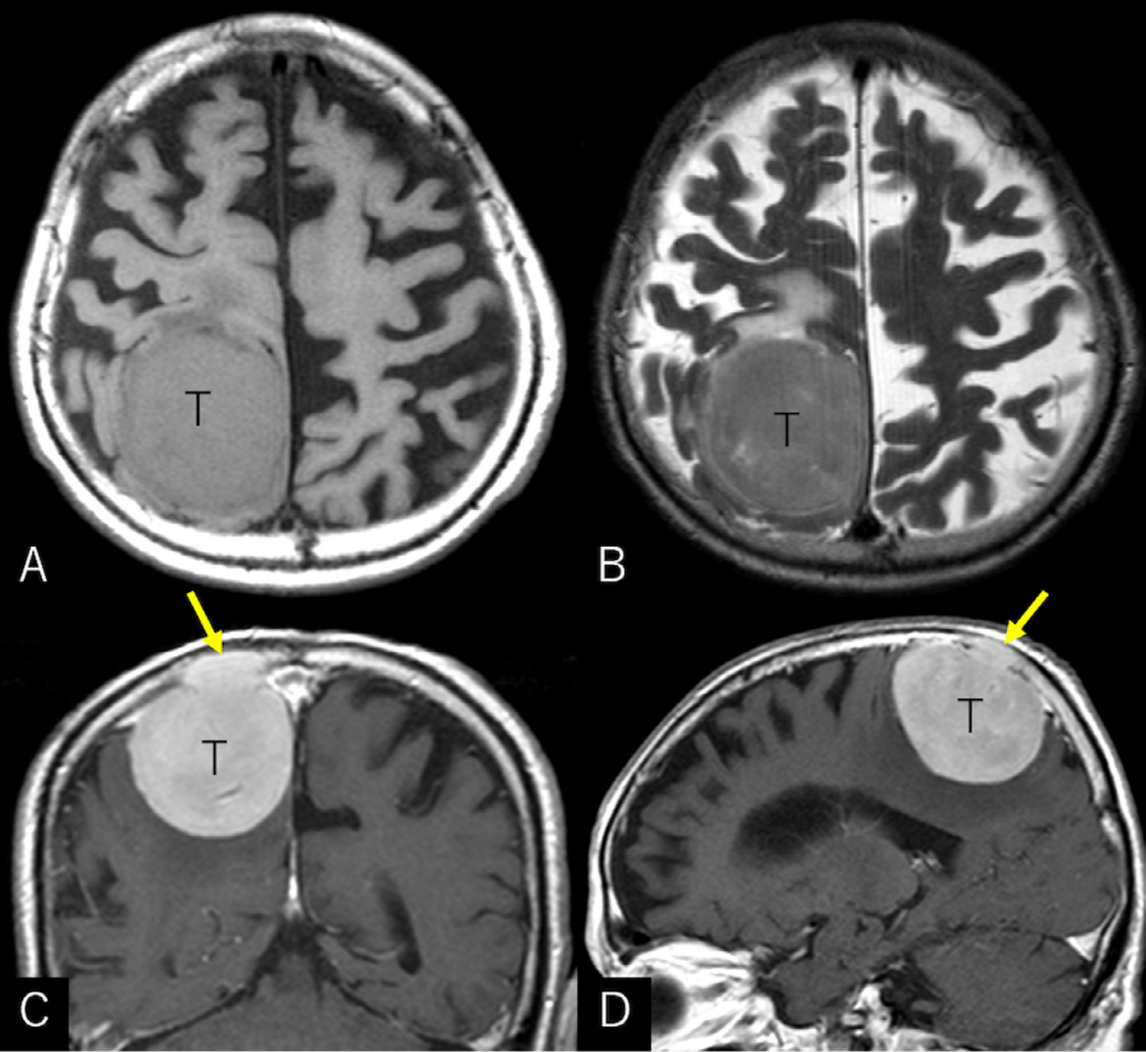
Axial T1- (***A***), T2-weighted (***B***), and postcontrast coronal (***C***) and sagittal (***D***) MRIs showing an intensely enhancing extra-axial mass. It appears isointense on T1- and hyperintense on T2-weighted sequences, respectively, broad-based on the convexity dura mater, and measuring 52 × 46 × 48 mm in maximal dimensions, with a portion upwardly protruding into the eroded skull.

**Fig. 3:**
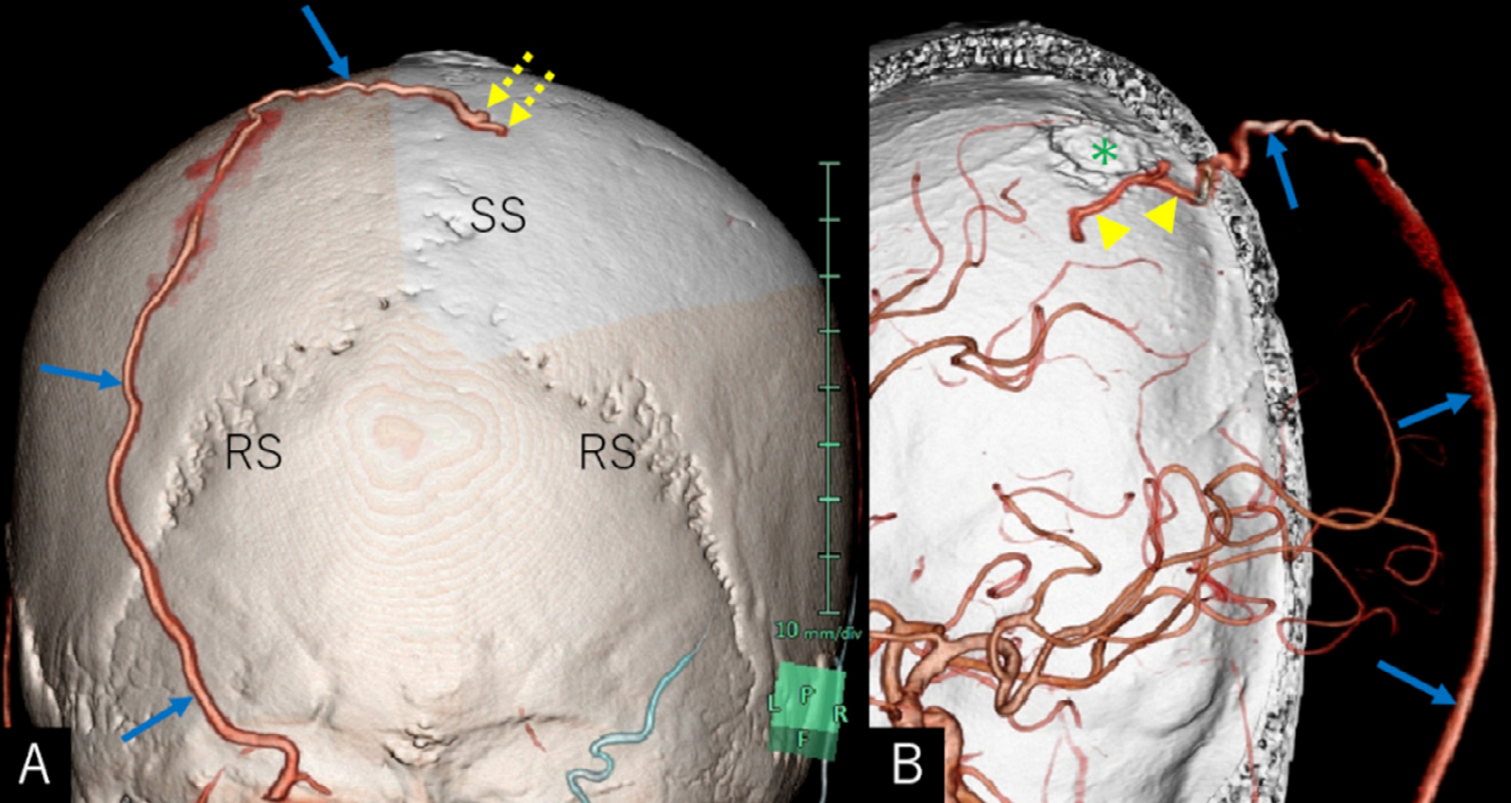
(***A***) Rear view of three-dimensional CT showing the ectatic left occipital artery (*arrows*) coursing upward, crossing the midline, entering into the right parietal foramina, and finally connecting with a dilated meningeal vessel that supplies the tumor (*dashed arrows*). (***B***) Interior view of the right parietal bone showing the occipital artery (*arrows*) that penetrates the parietal foramen and anastomoses with the meningeal vessel (*arrowhead*) adjacent to the bone erosion (*asterisk*) in the inner skull. RS: rhomboid suture; SS: sagittal suture.

**Fig. 4:**
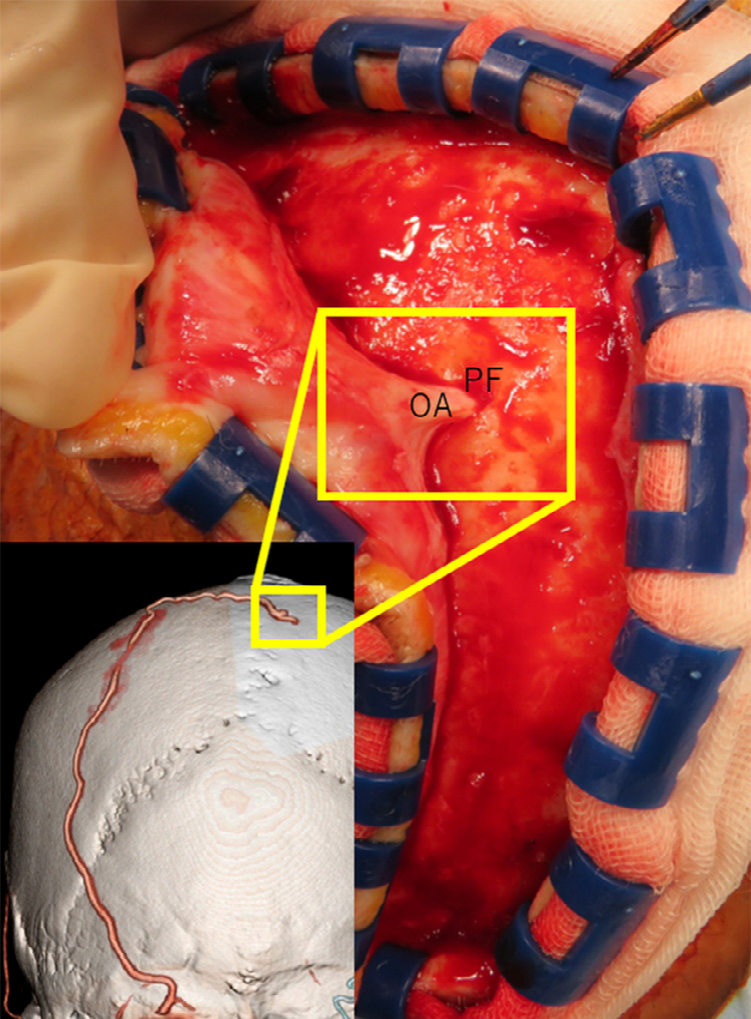
Intraoperative photo showing the occipital artery penetrating into the parietal foramen that has been exposed at the reflection of the scalp flap. OA: occipital artery; PF: parietal foramen.

**Fig. 5:**
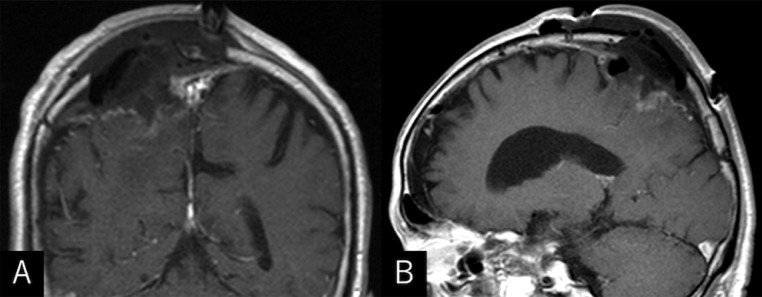
Postcontrast coronal (***A***) and sagittal (***B***) MRIs showing a total resection of the tumor.

**Fig. 6:**
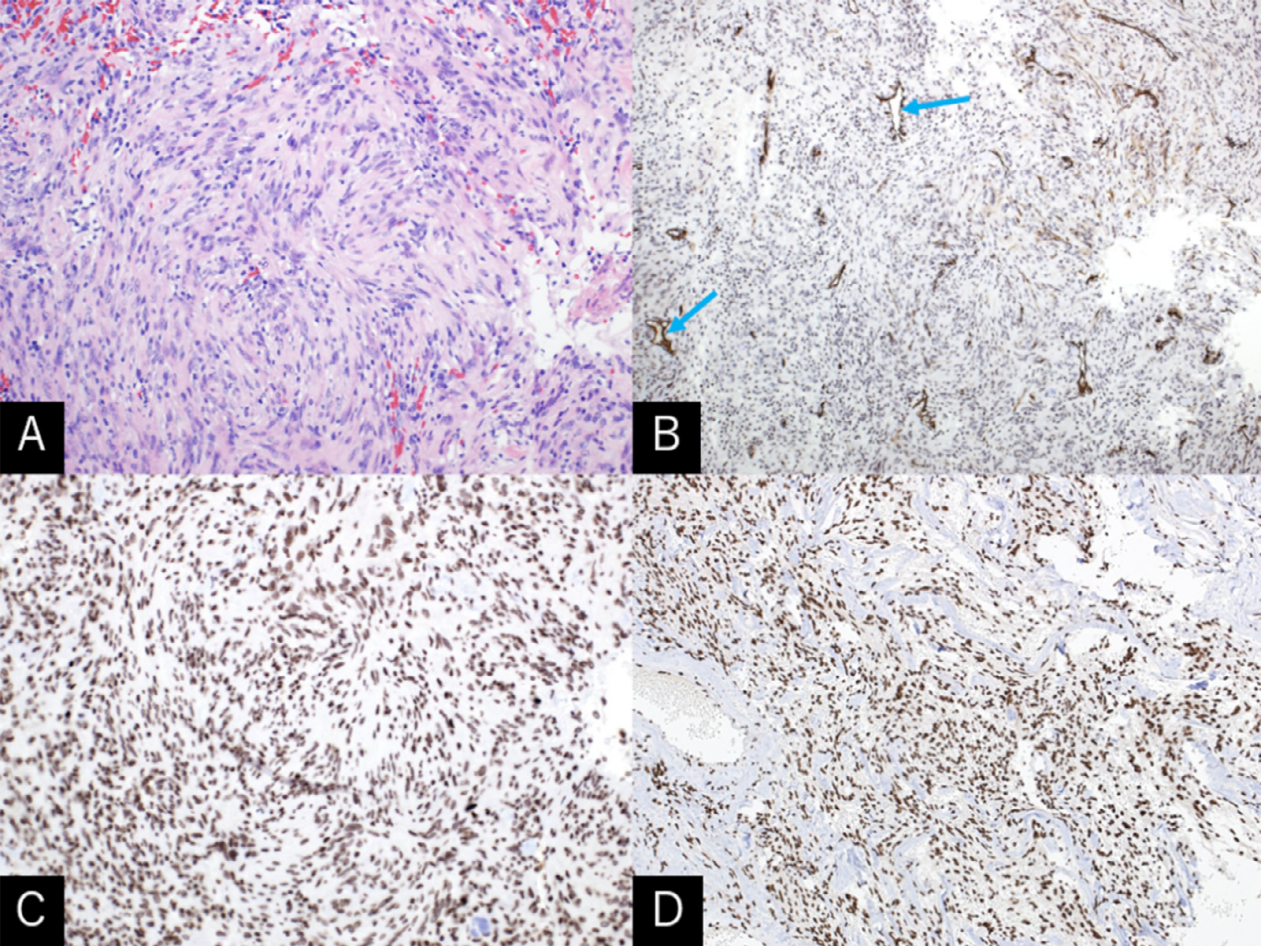
Photomicrographs of the resected specimen showing proliferation of oval- or spindle-shaped neoplastic cells arranged in storiform patterns (***A***). They show prominent angiogenesis occasionally presenting a staghorn appearance (***B***) with a MIB-1 index of 90% (***C***). The tumor cells are positively stained for STAT6 (***D***). ***A:*** hematoxylin and eosin stain, x200; ***B:*** CD34 stain, x100; ***C:*** Ki-67 stain, x200, ***D:*** STAT6 stain, x100.

An 86-year-old man sustained progressive motor weakness in the lower extremity for one month. At presentation, the patient was well oriented and presented with monoparesis (4/5 on the manual muscle test) in the left lower extremity. Cranial computed tomography (CT) revealed an isodense mass in the right parietal lobe with smooth-contoured focal erosion in the adjacent inner table. Neither intralesional calcification nor hyperostosis was observed in the adjacent skull (Fig. 1). The compressive tumor appeared extra-axial in location and isointense on T1- and hyperintense on T2-weighted magnetic resonance imaging (MRI), respectively, and intensely enhanced following intravenous gadolinium infusion. It was broad-based on the convexity dura mater and measured 52 × 46 × 48 mm in maximal dimensions, with a portion upwardly protruding into the eroded skull (Fig. 2). On three-dimensional CT angiography, the ectatic, contralateral left occipital artery was observed to course upward, crossing the midline, entering into the right parietal foramina, and finally connecting with a dilated meningeal vessel that supplied the tumor (Fig. 3A). The focal erosion in the inner parietal bone was formed adjacent to the feeding vessel (Fig. 3B). The patient underwent a tumor resection. Intraoperatively, the ectatic left occipital artery was coagulated and transected at the site entering the parietal foramina (Fig. 4). The dura mater overlying the tumor was eroded. The tumor, which was elastic hard and less vascular in consistency, was totally resected with sufficient margins of eroded dura mater (Fig. 5). Microscopic findings of the resected specimen showed proliferation of oval- or spindle-shaped neoplastic cells arranged in storiform patterns (Fig. 6A, B). Prominent angiogenesis occasionally presented a staghorn appearance, with an MIB-1 index of 90% (Fig. 6C). Immunohistochemical staining was positive for CD34 (not shown) and STAT6 (Fig. 6D), while negative for glial fibrillary acidic protein and epithelial membrane antigen. These findings were consistent with a World Health Organization grade III SFT/HPC. The patient was discharged on post-hospitalization day 30 with a modified Rankin Scale score of 2. Immediate adjuvant radiotherapy was not administered for him. Currently, the patient is under observation with periodic whole-body positron emission tomography/CT.

## Discussion

The prevalence of lesions mimicking meningiomas is estimated to be not negligible, suggesting the necessity of assuming a wide range of pathologies for varying prognoses and treatment strategies [1]. The present case was a high-grade SFT/HPC mimicking a meningioma that accompanied a distinct bone erosion. The tumor was exclusively fed by the transcranial blood supply from the contralateral occipital artery through the parietal foramina. The reason for contralateral supply might mainly be the dominance of bilateral occipital arteries, in addition to the location and distribution of the parietal foramina with variable anatomies [13,15]. In the present SFT/HPC, the accompanying bone erosion was located adjacent to the feeding vessel, while the tumor lacked hyperostosis and sunburst flow void as evident in the radiological findings. In addition, the dura mater covering the portion of the tumor with upward protrusion was eroded, which is an uncommon finding for meningiomas of the calvarial convexity. These findings were also identified in previously reported SFT/HPC cases [4,5,7,9].

The present SFT/HPC was a high-grade tumor that was likely to have a low overall prognosis due to a high risk of local recurrence and extracranial metastasis. In contrast, the patient was at an advanced age. Furthermore, a total resection was achieved by the initial surgery. Therefore, it was considered reasonable for the patient to be placed under observation without immediate adjuvant radiotherapy.

In conclusion, bone erosion and peripheral feeding vessels may be characteristic findings of intracranial SFT/HPCs. Careful interpretation of neuroimages could provide clues for differentiating an SFT/HPC masquerading as a meningioma from a true meningioma.

## Author Contributions

All the authors contributed equally to this study.

## References

[1] Nagai Yamaki V, de Souza Godoy LF, Alencar Bandeira G, Tavares Lucato L, Correa Lordelo G, Fontoura Solla DJ (2021). Dural-based lesions: is it a meningioma?. Neuroradiology.

[2] Chenhui Z, He G, Wu Z, Rong J, Ma F, Wang Z (2021). Intracranial solitary fibrous tumor/hemangiopericytoma: a clinical analysis of a series of 17 patients. Br J Neurosurg.

[3] Shin DW, Kim JH, Chong S, Song SW, Kim YH, Cho YH (2021). Intracranial solitary fibrous tumor/hemangiopericytoma: tumor reclassification and assessment of treatment outcome via the 2016 WHO classification. J Neurooncol.

[4] Chen Q, Chen XZ, Wang JM, Li SW, Jiang T, Dai JP. (2012). Intracranial hemangiopericytomas in children and adolescents: CT and MR imaging findings. AJNR Am J Neuroradiol.

[5] Chiechi MV, Smirniotopoulos JG, Mena H. (1996). Intracranial hemangiopericytomas: MR and CT features. AJNR Am J Neuroradiol.

[6] Liu X, Deng J, Sun Q, Xue C, Zhou Q, Huang X (2022). Differentiation of intracranial solitary fibrous tumor/hemangiopericytoma from atypical meningioma using apparent diffusion coefficient histogram analysis. Neurosurg Rev.

[7] Nishioka K, Tsutsumi S, Teramoto S, Nonaka S, Okura H, Suzuki T (2020). Atypical meningeal hemangiopericytoma presenting with punched-out calvarial erosion. Radiol Case Rep.

[8] Tan I, Soo MY, Ng T. (2001). Haemangiopericytoma of the trigeminal nerve. Australas Radiol.

[9] Tsutsumi S, Adachi S, Ishii H, Yasumoto Y. (2018). Atypical epidural hemangiopericytoma presenting with visual disturbance. Surg Neurol Int.

[10] Wang C, Xu Y, Xiao X, Zhang J, Zhou F, Zhao X. (2016). Role of intratumoral flow void signs in the differential diagnosis of intracranial solitary fibrous tumors and meningiomas. J Neuroradiol.

[11] Leipzig B, English J. (1984). Sphenoid wing meningioma occurring as a lateral orbital mass. Laryngoscope.

[12] Ruscalleda J, Feliciani M, Avila A, Castañer E, Guardia E, de Juan M. (1994). Neuroradiological features of intracranial and intraorbital meningeal haemangiopericytomas. Neuroradiology.

[13] de Souza Ferreira MR, Galvão APO, de Queiroz Lima PTMB, de Queiroz Lima AMB, Magalhães CP, Valença MM. (2021). The parietal foramen anatomy: studies using by skulls, cadaver and in vivo MRI. Surg Radiol Anat.

[14] Touré G, Méningaud JP, Vacher C. (2010). Arterial vascularization of occipital scalp: mapping of vascular cutaneous territories and surgical applications. Surg Radiol Anat.

[15] Tsutsumi S, Nonaka S, Ono H, Yasumoto Y. (2016). The extracranial to intracranial anastomotic channel through the parietal foramen: delineation with magnetic resonance imaging. Surg Radiol Anat.

[16] Yoshioka N, Rhoton AL, Abe H. (2006). Scalp to meningeal anastomosis in the parietal foramen. Neurosurgery.

